# Supraventricular Tachycardia (SVT) and Stroke: Should We Pump the Brakes on Cardioversion?

**DOI:** 10.7759/cureus.58193

**Published:** 2024-04-13

**Authors:** Andrew Hendrix, Thomas Eckert, Caroline Kerrison, Logan Carlyle, Anthony Yan

**Affiliations:** 1 Neurology, Prisma Health/University of South Carolina School of Medicine, Columbia, USA; 2 Internal Medicine, Prisma Health/University of South Carolina School of Medicine, Columbia, USA

**Keywords:** echocardiogram, stroke thrombectomy, cardioembolic strokes, chemical cardioversion, supraventricular tachycardia (svt), stroke

## Abstract

In the list of top 10 causes of death worldwide in 2019, stroke ranks number two, with a recent uptick in incidence involving younger adults. While common risk factors like tobacco use, hypertension, diabetes, and atrial fibrillation have been well studied, recent reports have also linked paroxysmal supraventricular tachycardia (PSVT) with strokes. This case highlights a rare presentation of a 25-year-old female who suffered an ischemic stroke shortly after undergoing chemical cardioversion for sustained SVT. To date, there are only three documented cases reporting an ischemic event following shortly after cardioversion of SVT, all confined to the pediatric population. Currently, there is limited evidence to guide the management of these complex patients. This case presents a valuable discussion regarding the futility or efficacy of imaging prior to cardioversion of SVT as well as furthers the conversation behind the theorized mechanisms linking PSVT and strokes.

## Introduction

Within the last 10 years, there has been a global surge in the incidence of stroke among young adults, typically defined as individuals aged 18 to 50 years [1,2]. Data from the US Nationwide Inpatient Sample spanning from 1995-2008 reveal a notable increase from 23% to 53% in ischemic strokes among those aged 15 to 44 years [3]. Shockingly, roughly one in ten strokes now occur in this younger demographic [3,4]. Contributing to this trend are increasingly high incidence and prevalence of risk factors such as hypertension, diabetes mellitus, dyslipidemia, and smoking [2,3]. Additionally, cardioembolic strokes, including those attributed to anomalies such as patent foramen ovale, constitute a significant portion of strokes in young patients, accounting for 47% of cases, while 11% have an undetermined etiology. Arterial lesions, frequently detected in critical arteries like the middle cerebral artery, internal carotid artery, and vertebrobasilar arteries, underscore the severity and complexity of these cases [2]. Emergent interventions for young stroke patients mirror those employed for older demographics, including physiologic management such as blood pressure, temperature, glucose, and oxygenation control, alongside thrombolysis when indicated [5]. Fortunately, the administration of intravenous thrombolysis in young stroke patients doesn't appear to heighten the risk of symptomatic intracerebral hemorrhage [2].

Supraventricular tachycardia (SVT) encompasses a spectrum of atrial and ventricular tachycardia characterized by rates exceeding 100 beats per minute at rest, primarily involving tissue originating at or above the bundle of His [6]. Evidence suggests that SVT affects approximately 2.29 per 1000 individuals in the general population, with roughly 89,000 new cases each year in the US [6]. Paroxysmal supraventricular tachycardia (PSVT) stands out as a subset of SVT marked by sudden onset and termination of a regular and rapid tachycardia [6]. While SVT accounts for a significant portion of emergency department (ED) and primary care physician visits, it is seldom the primary reason for hospital admission, underscoring its widespread impact on healthcare utilization [7]. Individuals with PSVT but no underlying cardiovascular disease tend to be younger (37 years vs 69 years), with faster rates when tachycardic compared to those with cardiovascular comorbidities [8]. Women face a twofold higher risk of developing PSVT, while individuals over 65 years old have more than five times the risk compared to younger counterparts [8]. Diagnosis of SVT relies heavily on the 12-lead electrocardiogram (ECG), which can aid in identifying the specific arrhythmia mechanism and guide subsequent treatment decisions. Treatment strategies for SVT, including pharmacotherapy, catheter ablation, or observation, must be tailored to the frequency and duration of SVT episodes and consider clinical manifestations and potential adverse consequences, such as cardiomyopathy. Adenosine emerges as an effective agent, supported by nonrandomized trials demonstrating high success rates in terminating SVT due to atrioventricular nodal reentrant tachycardia (AVNRT) or atrioventricular reentrant tachycardia (AVRT) in emergency or prehospital settings [6,9]. Pharmacological agents like verapamil, diltiazem, and adenosine yield favorable response rates ranging from 80% to 98% in stable SVT patients [6,9].

Cardioembolic stroke has primarily been associated with atrial fibrillation; however, the recognition of PSVT as a potential etiology for cardioembolic strokes has been relatively understated [10]. Recent studies are shedding light on this connection, revealing a substantial increase in the risk of ischemic stroke among patients with PSVT compared to those without [10-12]. The current nascent understanding is that PSVT could be an unrecognized stroke risk factor, potentially explaining a portion of strokes currently labeled as cryptogenic [13]. While a link between PSVT and stroke is becoming clear, there is very little literature showing cardioversion of PSVT itself as a risk factor for strokes. In fact, an extensive literature review yielded only three documented cases that discussed the occurrence of an ischemic event following shortly after cardioversion of PSVT, all confined to the pediatric population [14-16]. These cases suggest a possible association between PSVT cardioversion and embolic stroke, highlighting the need for further clinical attention and research into this potentially overlooked aspect of stroke etiology.

## Case presentation

**Figure 1:**
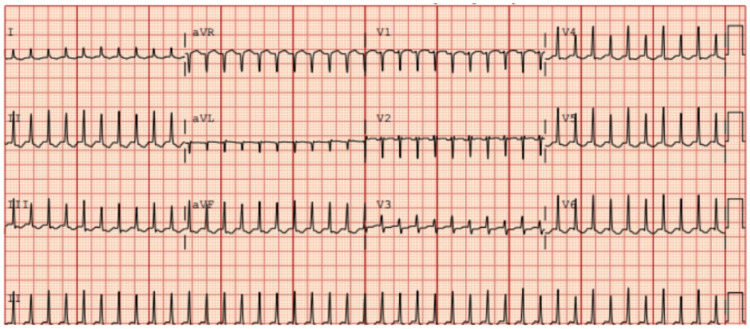
ECG demonstrating SVT prior to cardioversion. ECG = electrocardiogram, SVT = supraventricular tachycardia

A 25-year-old female presented to the ED due to cough, vomiting, and diarrhea for the past three days. Her past medical history was significant for SVT, which she reported began six months ago and had experienced three episodes in that time. In the ED, the patient was found to have a heart rate in the 250s as shown in Figure 1.

**Figure 2:**
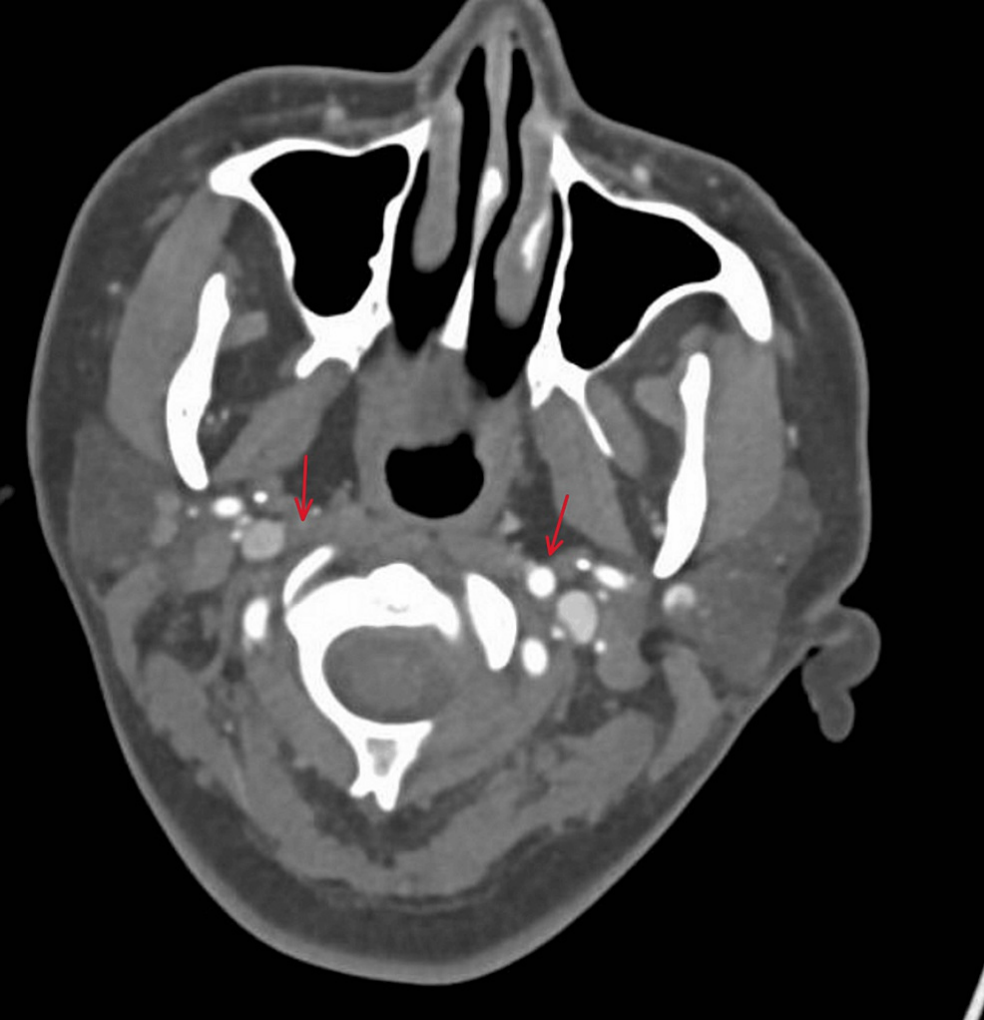
CTA of the head showing occlusion of RICA. CTA = computed tomography angiography, RICA = right internal carotid artery

Cardiology was consulted and chemically cardioverted with adenosine 6 mg. At the time of cardioversion, a point-of-care ultrasound was performed demonstrating preserved left ventricular function, no pericardial effusion, and no dilation of the left ventricle. However, a formal echocardiogram was deferred for fear of rapid decompensation as the patient’s heart rate had remained in the 250s for quite some time at this point. Roughly six hours later, the patient was being held in the ED pending admission when she developed acute onset left lower facial paralysis and dense left hemibody weakness and sensory loss. On initial stroke evaluation, NIHSS was 14 for partial left conjugate gaze palsy, left lower facial paralysis, flaccid left arm and leg with reported complete sensory loss to light touch, and sensory extinction on the left side. An emergency noncontrast CT head was negative for acute intracranial hemorrhage. Given the focal neurological signs on exam, the patient was consented for acute thrombolytic therapy. About 10 to 20 seconds after the thrombolytic bolus was administered, the patient was able to move her left arm, and later leg, against gravity. The patient was formally reevaluated and scored an NIHSS of 6 due to continued partial left conjugate gaze palsy, left lower facial paralysis, drift in the left arm and leg, and left-sided sensory neglect. CT angiogram of the head and neck showed an occlusion at the right ICA terminus, seen below in Figure 2, and the patient was consented for further neurointervention.

**Figure 3:**
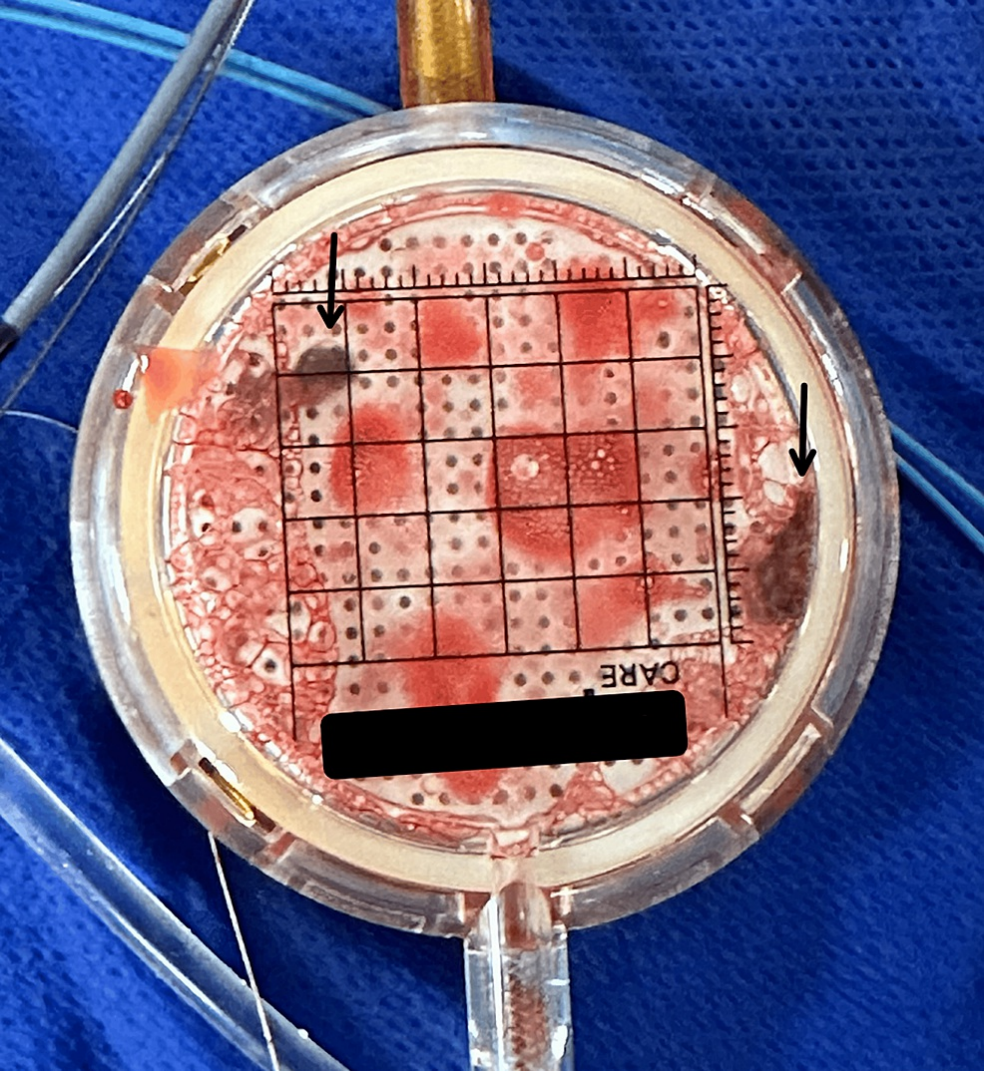
Pieces of thrombus collected after mechanical thrombectomy.

Mechanical thrombectomy was performed with TICI 3 reperfusion and post-operatively the patient demonstrated significant improvement, with recovery of near-full strength and sensation on post-stroke day 1. Figure 3 shows the pieces of thrombus collected after mechanical thrombectomy.

**Figure 4:**
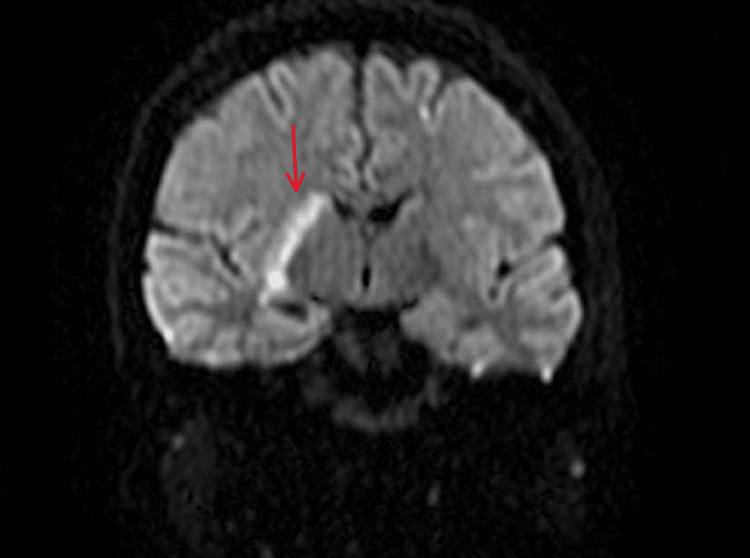
MRI of the brain showing diffusion restriction in the basal ganglia on the right side. MRI = magnetic resonance imaging

MRI brain showed areas of diffusion restriction predominantly in the right frontal lobe and basal ganglia, shown below in Figure 4, compatible with acute ischemic infarction.

There were additional smaller areas of ischemia within the posterior medial left frontal cortex and corona radiata. She was able to be transferred out of the neuroscience ICU on post-stroke day 4. During this time, lower extremity venous Doppler studies and a transesophageal echocardiogram (TEE) were performed to look for a stroke etiology, but both were unrevealing. Based on the clinical context, the vascular neurology team concluded the etiology of the stroke was most likely cardioembolic. The patient ultimately recovered well and at the time of discharge only had a slight weakness to her left side.

## Discussion

This is a rare case of a 25-year-old female who sustained an ischemic stroke shortly after chemical conversion for sustained SVT. As previously mentioned, only three documented cases have been reported in the literature that discussed the occurrence of an ischemic event following shortly after cardioversion of SVT, with all three confined to the pediatric population [14-16]. At the time, the authors suspected the etiology of the stroke to be embolic from a likely mural thrombus secondary to prolonged supraventricular tachycardia that was dislodged during cardioversion [16]. To date, the mechanism linking SVT and strokes has not been confirmed, though two main theories are commonly discussed in the literature. The first potential explanation involves the development of atrial cardiomyopathy due to persistent or recurring SVT. This process can cause apoptosis of myocardial cells and fibrosis, resulting in enduring structural alterations within the atria. Among these alterations, left atrial enlargement emerges as a prevalent sequela, which can induce blood flow stagnation and escalate the likelihood of thrombus formation [11-13]. The second rationale lies in the heightened susceptibility for subsequent atrial fibrillation as atrial fibrosis and left atrial enlargement act as pivotal catalysts in the onset of atrial fibrillation [11,17].

The risk of stroke following cardioversion of atrial fibrillation is well established and thus guidelines dictate when to obtain a TEE prior to cardioversion [18]. However, TEE is not found in the cardioversion protocol for SVT [19]. This unique case presents an interesting perspective on the futility or efficacy of TEE prior to SVT cardioversion. At the conclusion of the case, the vascular neurology team concluded the etiology of the stroke to be cardioembolic. This begs the question, should better imaging of the heart have been performed, and if so, would it have revealed a thrombus? If a thrombus was identified in the heart would this have changed management? Currently, there is little evidence in the literature to guide management in this situation. In discussion with the cardiology team, the stability of the patient must take priority as SVT can cause hemodynamic instability where the sedation and time for a procedure like TEE may result in poor outcomes. A TEE would also have poor quality due to the rapid heart rate. A transthoracic echocardiogram (TTE) requires less time and avoids sedation but does not have the spatial resolution to exclude a left atrial thrombus.

Nevertheless, it is important to note that the repercussions of stroke in young adults extend beyond acute management, with high rates of post-stroke unemployment and a persistently elevated long-term mortality risk, leading to significant decades of life lost [20]. Studies have shown that young adults who experience strokes are at a 2-3 times greater risk of unemployment even after eight years of follow-up and in the long term, face a notably higher mortality risk compared to the general population [20]. These findings underscore the urgency of addressing stroke prevention and management in younger populations to mitigate the growing burden of this condition.

## Conclusions

In light of the mounting occurrence of strokes among young adults, there is an undeniable urgency to uncover novel risk factors, such as SVT. This includes a deeper dive into mechanistic hypotheses linking SVT cardioversion and ischemic strokes through prospective studies integrating comprehensive methodologies, including transesophageal echocardiography, biomarker analysis, and advanced cardiac imaging. Such endeavors hold the potential to yield invaluable insights into the association between SVT and stroke, potentially paving the way for the evaluation and implementation of innovative management strategies.
